# A Molecular Interaction Map of *Klebsiella pneumoniae* and Its Human Host Reveals Potential Mechanisms of Host Cell Subversion

**DOI:** 10.3389/fmicb.2021.613067

**Published:** 2021-02-18

**Authors:** Deeya Saha, Sudip Kundu

**Affiliations:** Department of Biophysics, Molecular Biology and Bioinformatics, Faculty of Science, University of Calcutta, Kolkata, India

**Keywords:** pathogen–host interaction, biomolecular network, host cell hijacking, p53 signaling cascade, immune surveillance machinery

## Abstract

*Klebsiella pneumoniae* is a leading cause of pneumonia and septicemia across the world. The rapid emergence of multidrug-resistant *K. pneumoniae* strains necessitates the discovery of effective drugs against this notorious pathogen. However, there is a dearth of knowledge on the mechanisms by which this deadly pathogen subverts host cellular machinery. To fill this knowledge gap, our study attempts to identify the potential mechanisms of host cell subversion by building a *K. pneumoniae*–human interactome based on rigorous computational methodology. The putative host targets inferred from the predicted interactome were found to be functionally enriched in the host’s immune surveillance system and allied functions like apoptosis, hypoxia, etc. A multifunctionality-based scoring system revealed P53 as the most multifunctional protein among host targets accompanied by HIF1A and STAT1. Moreover, mining of host protein–protein interaction (PPI) network revealed that host targets interact among themselves to form a network (TTPPI), where P53 and CDC5L occupy a central position. The TTPPI is composed of several inter complex interactions which indicate that *K. pneumoniae* might disrupt functional coordination between these protein complexes through targeting of P53 and CDC5L. Furthermore, we identified four pivotal *K. pneumoniae-*targeted transcription factors (TTFs) that are part of TTPPI and are involved in generating host’s transcriptional response to *K. pneumoniae-*mediated sepsis. In a nutshell, our study identifies some of the pivotal molecular targets of *K. pneumoniae* which primarily correlate to the physiological response of host during *K. pneumoniae-*mediated sepsis.

## Introduction

*Klebsiella pneumoniae* is a nosocomial pathogen that can cause a wide repertoire of infections, including pneumonia (Prince et al., 1997), sepsis (Cryz et al., 1984; Karabinis et al., 2004), meningitis (Saccente, 1999; Wu et al., 2009), bacteremia (Stotka and Rupp, 1991; Ko et al., 2002), urinary tract infection (UTI) (Stotka and Rupp, 1991), and pyogenic liver abscesses (Chung et al., 2007; Wu et al., 2009; Siu et al., 2012; Lin et al., 2013). *K. pneumoniae* accounts for 5–20% of bacterial sepsis worldwide which is often associated with poor prognosis and severe outcomes including death (Nordmann et al., 2009; Eddens and Kolls, 2012). *K. pneumoniae* was originally found to infect patients with a compromised immune system and with other comorbidities like diabetes, cancer, and organ transplantation (Garcia de la Torre et al., 1985; Lin et al., 2013; Lee et al., 2017). However, in recent years, there is an increased emergence of hypervirulent and multidrug-resistant strains of *K. pneumoniae* that has the potential to cause infection even in the healthy, immune-sufficient individuals (Kader et al., 2007; Lin et al., 2012; Olaitan et al., 2014). The emergence of hypervirulent and antibiotic-resistant strains of *K. pneumoniae* and their worldwide spread necessitate more comprehensive research on *K. pneumoniae* that could shed light into the molecular details of *K. pneumoniae-*mediated pathophysiology.

There are mainly four types of virulence factors in *K. pneumoniae*: (i) capsule (Struve and Krogfelt, 2003; Schembri et al., 2005; Evrard et al., 2010; Rendueles, 2020), (ii) fimbriae (Murphy et al., 2013; Lin et al., 2017), (iii) lipopolysaccharides (Kamaladevi and Balamurugan, 2016), and (iv) siderophores (Holden et al., 2016, 2018; Zhang et al., 2017) that have been well studied and are important for virulence in infection models. However, there are several genes in the *K. pneumoniae* genome that remain poorly characterized and might have potential roles in virulence (Pranavathiyani et al., 2020). A previous study has identified some of the *in vivo* fitness genes in *K. pneumoniae* which are required by this pathogen to survive and adapt to the host environment (Bachman et al., 2015). These fitness genes include genes responsible for the branched-chain amino acid synthesis and serum resistance.

Although fitness and virulence are related to each other, they are two distinct entities. Fitness genes are defined as the genes in a pathogen whose deletion impedes its growth within a host, whereas virulence factors are proteins whose deletion reduces its pathogenicity (Subashchandrabose and Mobley, 2015). To be a successful virulence factor, a pathogen protein must have physical interactions with host cell biomolecular machinery including proteins (Casadevall and Pirofski, 2001; Wallqvist et al., 2015). This facilitates host cell subversion by the virulence factors of the pathogen (Crua Asensio et al., 2017). However, often a subset of fitness imparting genes act as virulence factors (Subashchandrabose and Mobley, 2015), for instance, in uropathogenic *Escherichia coli* (UPEC) the type IV pilus which is a known virulence factor and is involved in imparting fitness to the pathogen (Subashchandrabose and Mobley, 2015). According to Subashchandrabose and Mobley (2015), type IV pilus confers some selective advantages to UPEC and thus helps it to survive in the urinary tract of the host. In this connection, Crua Asensio et al. (2017) advocate that fitness of a pathogen is determined by the successful interaction of the pathogen with its host. They also showed that the higher the fitness of a pathogen protein, the more it tends to interact with host cell proteins. To this end, there is no protein interaction data available between the fitness genes of *K. pneumoniae* and proteins in the human host. Therefore, the complete molecular details of host-cell subversion mechanisms by *K. pneumoniae* indeed remain unidentified.

Thus, a *K. pneumoniae*-human protein–protein interaction (KHPPI) network could not only help us to identify the key virulence and fitness-related factors in *K. pneumoniae* but also would give us a mechanistic insight into the putative functions and molecular interactions perturbed by *K. pneumoniae* during its infection. However, previous large-scale systematic yeast two-hybrid (Y2H) studies focussed on virus–host PPI networks (Calderwood et al., 2007; Simonis et al., 2012; Luo et al., 2016; Farooq, Q. ul et al., 2020). Bacterial–host PPI networks are largely unidentified with exception to few studies (Dyer et al., 2010; Memiševiæ et al., 2015). Also, for a bacterial pathogen, which has a large genome constituting thousands or more genes, identification of virulence factors and large-scale mapping of their host protein interactors demands both time and a lot of effort. In this context, computational strategies to identify putative host targets by pathogen play a pivotal role (Arnold et al., 2012).

Among the several computational strategies used to predict pathogen–host interaction network, interolog-based predictions are fairly robust and have been widely used previously to predict the PPI landscape between the many unknown pathogens of interest and their host cellular proteins (Kumar and Nanduri, 2010; Rapanoel et al., 2013). This method uses previously characterized PPI data from other well-studied species pairs (a pathogen and a host) and inferred PPIs for the unknown pathogens of interest–host of interest species pairs by orthology-based deductions. The limitation of this method is that it does not give any information on PPIs of bacterial pathogen proteins that are strain-specific or are poorly conserved across phylogenetic lineages. Moreover, interolog-based approaches rely on known pathogen–host interactome which are previously inferred by experiments; it cannot predict novel interactions (Wang et al., 2020). In addition, with increase in evolutionary distances between species, processes like neofunctionalization occur and this makes the interolog-based approaches error-prone due to difficulty in ortholog prediction based on poor sequence similarity (Eichinger et al., 2016). In this context, motif-based and protein structure-based inferences of host–pathogen interactions show great promise (Via et al., 2015; Sámano-Sánchez and Gibson, 2020). Short linear motifs act as potential site for pathogen–host interactions and these linear motifs also show convergent evolution which cannot be captured through interolog approach (Zanzoni et al., 2017). However, lack of well-reviewed protein structures in *K. pneumoniae* limits application of structure-based inferences of protein–protein interactions (PPIs) between *K. pneumoniae* and its human host.

Here, in this study, we first designed an interolog-based three-step computational strategy where we have integrated multiple high throughput pathogen–host interaction datasets and used them as a template to derive a comprehensive molecular interaction map between *K. pneumoniae* and the human host. This interaction map was then further filtered based on *in vivo* fitness, Gene Ontology (GO)-term similarity, and secretion propensities of *K. pneumoniae* proteins. Subsequent network-based downstream analyses with *K. pneumoniae-*targeted host proteins were executed where we integrated host’s high throughput genetic, proteomic, and transcriptomic dataset to identify influential molecular targets shedding light into the mechanism of host cell subversion. We observed predicted host targets to be highly enriched with proteins that are an integral part of the host’s immune surveillance machinery. We identified a key influential molecular target, P53 which could have a profound role in *K. pneumoniae-*mediated disease pathogenesis. We observed that multifunctional protein P53 mediates several inter-complex interactions. Therefore, targeting P53 and its interactome could be an effective strategy by *K. pneumoniae* to disrupt coordination between protein complexes. Moreover, we identified four key transcription factors (TFs) that were observed to be differentially expressed in *K. pneumoniae* positive sepsis patients. These four TFs (HIF1A, STAT1, ETS2, and EGR1) were found to be interactors of P53 and could potentially regulate several downstream DEGs in sepsis patients. Together, our results, based on computational predictions, indicate that targeting P53 and its interactors could have a profound effect on the host’s physiology during *K. pneumoniae-*mediated infection. However, these results are based on *in silico* strategies and need to be validated experimentally.

## Materials and Methods

### Retrieval of Sequences of *Klebsiella pneumoniae* KPPR1 Strains

The protein sequences and annotations of *K. pneumoniae* KPPR1 strain were retrieved from NCBI Refseq database (O’Leary et al., 2016) using the following link: https://ftp.ncbi.nlm.nih.gov/genomes/refseq/bacteria/. The sequences thus retrieved were further screened. Hypothetical proteins and incomplete protein sequences were eliminated from the analysis.

### Identification of Interologs

Host–Pathogen Interaction Database (HPIDB) version 3.0^1^ (Kumar and Nanduri, 2010) was used to retrieve all experimentally established pathogen–host PPI (PHPPI) data. Only those PPIs that were inferred from experiments such as Y2H, co-immuno-precipitation, and other experimentally robust protocols were considered to be the template for predicting KHPPI. NCBI reciprocal BlastP (Altschul et al., 1990) was used to search for homologous proteins in the proteome of *K. pneumoniae* KPPR1 strain and *Homo sapiens*. We used a stringent cut-off of E-value less than 10^–10^, 90% query coverage, and 50% sequence similarity for blast homology search. To find out the potential KHPPIs that play a significant role in *K. pneumoniae* virulence, we chose *K. pneumoniae* fitness factor as a screening constraint of KHPPI. The KHPPI was screened and only those *K. pneumoniae* proteins were chosen which has a high fitness value in the mouse model. The *K. pneumoniae* KPPR1 fitness data were retrieved from Bachman et al. (2015). Genes with high fitness values are those that are essential for survival within the host. Here, we chose fitness ratio > 2.0 and cedar *P*-value = 0 (confirmed by cedar analysis) as a cut-off. The entire set of predicted KHPPI (both filtered and raw) along with fitness data are provided in Supplementary Tables 1, 2, respectively.

### GO Term Similarity Analysis Between Inferred Homologs

We first downloaded the functional annotations of each member of the homolog pairs from uniport database (Bateman et al., 2017). The functional annotations were based on GO terms. We considered a given homolog pair to be functionally similar if they shared at least one common GO term between them. To calculate the significance level of the observed proportion of functionally similar homolog pairs in our dataset, we compared the functional similarity of real homolog pairs to that of randomized non-homologous protein pairs. A list of non-homologous protein pairs (did not yield a significant hit when reciprocal protein blast search was conducted against *K. pneumoniae.* A significant reciprocal blast hit is defined as E-value 10^–10^, query coverage ≥ 90%, sequence identity > 50%) was generated for each of pathogen proteins. Supplementary Table 3 shows the pathogen species used to generate the list of non-homologous protein pairs along with the number of non-homologous proteins against each of these pathogen species. These were added up to a total of 11,357 non-homologous gene pairs between *K. pneumoniae* genome and other pathogen genomes, respectively. We randomly chose 183 (because there were 183 *K. pneumoniae* homolog pairs that yielded filtered KHPPI) non-homologous gene pairs for 1000 times and observed their functional similarity. The observed proportion of functionally similar gene pairs in homologs was higher compared to non-homologs in each of the 1000 times (*P* = 3.43 × 10^–5^; Fisher’s exact test).

### Prediction of Secretion Propensity of *K. pneumoniae* Proteins

To assess the secretion propensity of the 183 *K. pneumoniae* proteins obtained herein, we combined two prediction strategies, i.e., (i) BastionHub (Wang et al., 2020) and (ii) EffectiveDB (Eichinger et al., 2016). We have used BastionX (included in the BastionHub suite) to predict the secretion propensity of each of the *K. pneumoniae* proteins. BastionX utilizes a machine-learning-based approach to predict the secretion propensity of the given proteins. It can predict substrates of Type I–VI secretion systems in Gram-negative bacteria. The predicted results of BastionX are provided in Supplementary Table 4. On the other hand, EffectiveDB predicts substrate of Type III, IV, and VI secretion systems through detection of secretion peptides. It can also predict the secretion propensity of a given protein through detection of Eukaryotic-Like Domains (ELDs) in the protein sequences. ELDs are widely present in virulence effectors of Gram-negative bacteria (Gomez-Valero et al., 2019; Mondino et al., 2020). Hence, an enrichment of ELDs in a given protein sequence indicates high probability of it to be secreted.

### Function Enrichment Analyses

ClusterProfiler version 3.04 (Yu et al., 2012), an R package, was used to conduct GO analysis as well as KEGG pathways enrichment analysis. The ClusterProfiler uses fisher’s exact test to identify significant GO terms or KEGG pathways. Benjamini–Hochberg test was performed to determine the adjusted *P*-values of each GO terms and KEGG pathways. Fold enrichment values were calculated as mentioned below:

$$\mathrm{Fold}\mathrm{enrichment}=\frac{m/n}{M/N}$$

*M* = total no. of genes annotated with the given enriched KEGG pathways or GO terms in human proteome; *N* = total no. of genes functionally annotated in *H. sapiens* proteome; *m* = total no. of genes annotated with the enriched KEGG pathways or GO terms in the host target gene set; and *n* = total no. of genes in the host target gene set.

All plots related to function enrichment were generated using the seaborn package of Python v3.7. The details of the results of GO and KEGG pathways enrichment analysis are provided in Supplementary Tables 5, 6, respectively. Each GO ID or KEGG pathways were assigned with a functional domain. This functional domain assignment was done very carefully. GO IDs or KEGG pathways that were similar were assigned the same functional domain. For instance, GO:0071346 and GO:0071349 (Supplementary Table 5) both refer to cytokine production, hence their functional domain is the immune response.

### PPI Network Analyses

The human PPI network was constructed based on PPI data from two resources: (i) STRING version 11^2^ (Szklarczyk et al., 2019) and (ii) BioGRID version 3.5^3^ (Stark, 2006). We selected binary PPIs with STRING cumulative score 900 and above. STRING computes score of each PPI based on the following evidences fusion, co-occurrence, coexpression, experimental, database, and text mining and generates a score combining the individual scores of these evidences. We used the following criteria to screen the binary PPI inferred from STRING. (1) We excluded PPIs which were not supported by experimental evidence and included only those PPIs that were supported with an experimental evidence. (2) A PPI is excluded from the dataset if it was supported by only other sources of evidences like coexpression, co-occurrence, and fusion and not by experimental evidences. (3) Apart from (1) and (2), we retained only those PPIs in our dataset which had a cumulative score ≥ 900. When we incorporated PPI data from BioGRID, we made sure that the PPI concerned was based on physical PPI inferred from direct experimental systems like Y2H. Such high confident PPIs were used to build up the raw human PPI network (Supplementary Table 7). Using the following stringency cut-off, we gathered a human PPI network comprising of 8823 proteins connected through 45,215 interactions.

### Construction, Visualization, and Calculation of Network Statistics

Cytoscape version 3.7.1 (Shannon et al., 2003) was used for construction, visualization, and calculation of topological parameters of the inferred PPI network.

### Integration of Protein Complex Data to PPI Data

Human protein complex information was retrieved from the core protein complex dataset of CORUM^4^ database (Ruepp et al., 2009). The entire dataset of proteins and the protein complex in which they participate is provided in Supplementary Table 8. Degree of intermodularity (*DI*) was calculated as follows:

DI=⋂CjCi⋃CjCi

where *i* and *j* are two physically interacting proteins; *Ci* and *Cj* are protein complexes in which *i* and *j* participate. The *DI* ranges from 0 to 1. *DI* of 0 indicates that the two proteins do not share any protein complex between them and therefore are inter-complex or intermodular. This indicates that the protein pairs involved in interaction might be connecting or coordinating two or multiple functional protein complexes. If *DI* value rises above 0 but is still less than 1, it indicates that the protein pairs involved in interactions have both shared and unshared protein complexes between them. *DI* value of 1 indicates that there are no unshared protein complexes between a given pair of proteins or in other words both proteins A and B participate in the same protein complex or complexes (Figure 3A). *DI* value of 1 indicates strictly intramodular interactions.

### Module Analysis of Weighted Network

MCL clustering in ClusterMaker version 2.0 (Morris et al., 2011) (a Cytoscape app) was used for module analysis of the function–function interaction network (FFIN). Each edge in the FFIN was assigned a weight which is sum of the number of unique inter-complex PPIs connecting the respective functions.

### Functional Analysis of Inferred Networks

The Biological Networks Gene Ontology tools (BiNGO) (Maere et al., 2005), a Cytoscape plugin was used to determine enriched functions in the inferred PPI network. Plots were generated using Python and R packages.

### Regulatory Network Analysis

The human regulatory network was retrieved from the RegNetwork database^5^ (Liu et al., 2015) and the Transcriptional Regulatory Relationships Unraveled by Sentence-based Text mining (TRRUST) database version 2.0^6^ (Han et al., 2018). Only experimentally inferred TF-TG interactions were considered for building up the regulatory network. The human regulatory network thus built is provided in Supplementary Table 9.

### Gene Expression Analysis

Microarray dataset was downloaded from the Gene Expression Omnibus (GEO) database (Barrett et al., 2013) for five patients suffering from sepsis with blood culture positive for *K. pneumoniae* in a Thailand hospital (GSE69528) (Pankla et al., 2009). The above-mentioned expression dataset was analyzed using GEO2R (Barrett et al., 2013). Only those genes that had adjusted *P*-value (Benjamini–Hochberg) < 0.05 and fold change > 2.0 in the *t*-test were considered as differentially expressed genes (DEGs). The above-mentioned cut-off yielded a total of 1413 DEGs (Supplementary Table 10).

### Statistical Analyses

All statistical tests used in this study including Mann–Whitney *U*-tests and Fisher’s exact tests were conducted using R version 4.1.2. Randomization tests were done using in-house R scripts.

## Results

### Identification of Potential *K. pneumoniae*-Targeted Host Proteins

**FIGURE 1:**
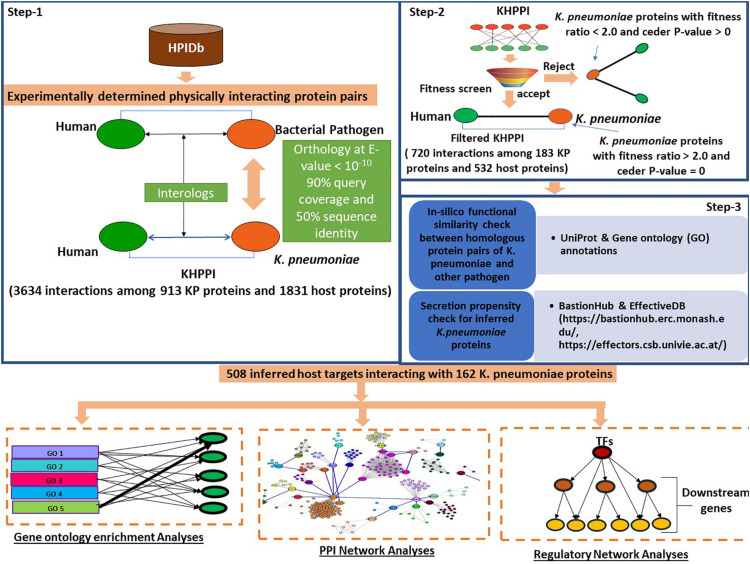
Schematic representation of the workflow followed to predict *K. pneumoniae-*targeted host proteins and subsequent downstream functional analyses with the *K. pneumoniae* predictome. To predict *K. pneumoniae-*targeted host proteins, we devised a three-step computational strategy. In the first step using HPIDb (an exhaustive repository of experimentally determined host–pathogen interaction datasets), we identified potential interologs between *K. pneumoniae* and humans at e-value 10^– 10^, 90% query coverage, and 50% sequence identity. Further, the *K. pneumoniae* proteins predicted to interact with host proteins were screened based on their fitness in mouse (Fitness data derived from Bachman et al., 2015). Those *K. pneumoniae* proteins with a fitness value > 2.0 were retained in step 3; an *in silico* GO term similarity check along with secretion propensity checks was carried out. The final KHPPI thus filtered were subjected to further downstream network analysis.

At first, we aimed to identify the potential host proteins that interact with *K. pneumoniae* proteins. To predict this cross-species PPI, we have designed a three-step computational strategy (Figure 1).

In the first step, we built a potential *K. pneumoniae*–human cross-species PPI (KHPPI) based on the interolog approach. We assembled high-throughput bacterial pathogen–human host PPI data from HPIDB and used them as templates to identify potential KHPPIs through stringent homology search against *K. pneumoniae* proteome. Since the host species in the template PPI was human, we carried out the homology search between pathogen proteins and *K. pneumoniae* proteins only. Upon stringent reciprocal blast search (E-value ≤ 10^–10^, query coverage ≥ 90%, and sequence identity > 50%) of the template PPI against *K. pneumoniae* proteome, we were able to identify 3634 putative PPIs between 913 *K. pneumoniae* proteins and 1831 human proteins (Supplementary Table 1).

The second step of our computational workflow includes the screening of 913 *K. pneumoniae* proteins which were predicted to interact with 1831 human proteins in the previous step. We here adopted a screening technique based on fitness ratio in published mouse models (Bachman et al., 2015). The rationale behind considering fitness as a factor for screening *K. pneumoniae* proteins is the relationship between the fitness of pathogen proteins and its tendency to interact with host proteins (Crua Asensio et al., 2017). It has been previously shown by Crua Asensio et al. (2017) that pathogen proteins whose deletion incur a high fitness cost on the survival of pathogen within the host play an instrumental role in interaction with host proteins. So, we have deployed fitness as a factor to screen the 913 *K. pneumoniae* proteins obtained in step 1. Bachman et al. (2015) quantified fitness as a ratio of bacterial inoculum mutated and injected into a mouse by bacterium inoculum obtained from mouse lung. They emphasized on 332 *K. pneumoniae* proteins that had a fitness ratio ≥ 2.0 and a cedar *P*-value of 0 (Supplementary Table 2). This implies that the deletions of these individual 332 genes might cause a twofold fitness defect in *K. pneumoniae* within the host. We noticed that there were 183 *K. pneumoniae* proteins which were common to both the subset of 332 fitness factor proteins and 913 *K. pneumoniae* proteins involved in the interaction with the host (identified in the previous step). These 183 *K. pneumoniae* proteins were observed to be interacting with 532 human proteins yielding a total of 720 PPIs (Supplementary Table 1).

In the third step, we tried to show that *K. pneumoniae* proteins involved in interactions with the human host proteins are indeed (1) true homologs of previously known pathogen proteins in the template PPI and that these (2) *K. pneumoniae* proteins are secretory proteins localizing in pathogen host interface to mediate potential interactions with the host proteins. To achieve our first aim, we conducted an *in silico* GO term similarity analysis where we retrieved the functional annotations of the proteins that are homologous between *K. pneumoniae* and other pathogen species from uniprot. We noticed that 152 out of 183 *K. pneumoniae* (∼83%) proteins show functional similarity with the corresponding homolog in other pathogen species, i.e., they shared at least one GO term between them. The rest of the 17% of the homologous had unknown functions. This percentage of 83% was indeed higher than that expected by chance (Odd’s ratio = 2.21, *P*-value = 2.76 × 10^–5^; Fisher’s exact test) (see section “Materials and Methods”). Thus, it could be inferred that the 183 homologs of *K. pneumoniae* that comprise the screened or filtered KHPPIs were probably enriched in true functional homologs. Since very few (17%) of the homolog pairs have unknown functions, we did not eliminate them as elimination of these pairs with unknown functions could lead to elimination of potential host interactors from our dataset.

Next, we tried to show that *K. pneumoniae* proteins identified to be interacting with human host proteins through interolog approach are secretory proteins that act like effector molecules in *K. pneumoniae.* For this, we predicted the secretion propensity of each of the 183 *K. pneumoniae* proteins by combining two independent prediction tools BastionHub and EffectiveDB (Eichinger et al., 2016; Wang et al., 2020). The results of these two prediction servers were summarized in Supplementary Table 4. In total, we found 162 *K. pneumoniae* proteins that were found to have putative secretory property due to possession of signal peptides and or ELDs. There were 3, 24, 3, and 1 proteins belonging to Type II, Type III, Type IV, and Type VI secretion system, respectively (Supplementary Table 4). There were 31 *K. pneumoniae* proteins out of 183 that participated in secretion systems and therefore are highly secretory (Supplementary Table 4). Apart from this, effectiveELD yielded 131 *K. pneumoniae* proteins that were enriched in ELDs and thus could behave like effectors. This again shows that > 85% of the inferred *K. pneumoniae* proteins are secretory in nature and might localize in the host–pathogen interface to facilitate interaction with the host proteins. However, 21 proteins were there that do not show any secretory property. We therefore eliminated these 21 proteins from our analysis. This led to a total of 671 interactions between 162 *K. pneumoniae* and 508 host interactors. These 508 host target proteins were subjected to further downstream analysis to provide functional implications of these interactions in the context of host cell physiology.

### *K. pneumoniae-*Targeted Host Proteins Are Enriched in Tightly Interlinked, Overlapping and Immune-Related Functions

In this section, we aimed to identify underlying metabolic pathways and biological processes that are targeted by *K. pneumoniae*. The 508 host targets inferred in the previous section were subjected to detailed function enrichment analyses using GO terms and KEGG pathway information. GO terms were further subdivided into three categories: (i) GO biological processes (GO_bp_), (ii) GO molecular functions (GO_mf_), and (iii) GO cellular compartment (GO_cc_). The results of this enrichment analyses are delineated below.

#### GO_bp_ Enrichment Analysis

**FIGURE 2:**
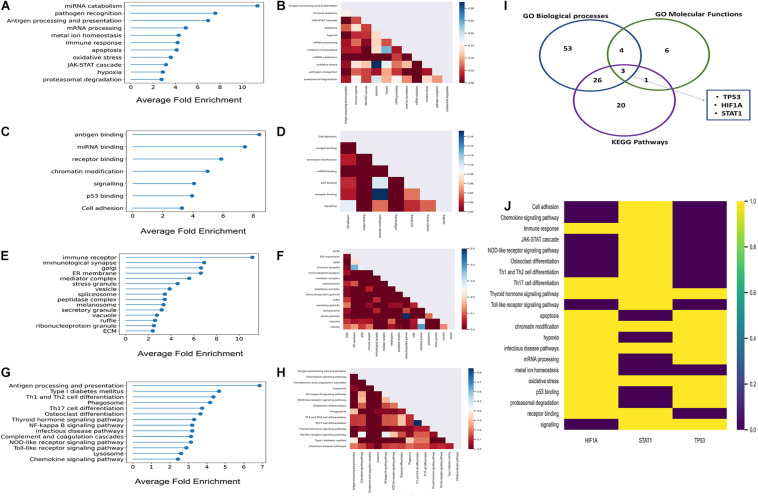
Results of the GO enrichment and KEGG pathways enrichment analyses. **(A,C,E,G)** Lollipop plots of GO_bp_, GO_mf_, GO_cc_, and KEGG pathways enrichments, respectively. Here, *X*-axis represents fold enrichment of each of the enriched functional domains derived from enriched GO terms and KEGG pathways. **(B,D,F,H)** Partial heatmaps showing the number of host targets shared between each of the enriched GO_bp_, GO_mf_, GO_cc_, and KEGG pathways. **(I)** Venn diagram where the blue, green, and purple circles represent GO_bp_, GO_mf_, and KEGG pathways, respectively. The number within each circle represents the number of multifunctional host targets in the respective functional categories. Five multifunctional proteins were common to three of the functional categories; these were P53, HIF1A, and STAT1. **(J)** Heatmap showing the aforementioned highly multifunctional host targets and the functions in which they participate.

There were 136 statistically significant GO_bp_ terms (*P* < 0.05; Fisher’s exact test, multiple test correction using Benjamini–Hochberg method) which were further grouped into 11 functional categories (Supplementary Table 5). The average fold enrichment values for each of these 11 functional categories, ranked in ascending order are depicted in Figure 2A. The number of shared proteins between each of these functional categories is represented in Figure 2B. GO_bp_ terms related to cytokine production, antigen processing and presentation, and immune receptor-mediated signaling are found to be highly enriched among host targets. GO_bp_ terms like hypoxia, response to oxidative stress, JAK-STAT cascade, ubiquitin-mediated protein degradation, and apoptosis which might have a direct impact on immune surveillance machinery are also found to be enriched among host targets.

#### GO_mf_ Enrichment Analysis

We identified 18 statistically significant GO_mf_ terms (*P* < 0.05; Fisher’s exact test, multiple test correction using Benjamini–Hochberg method). These GO_mf_ terms were further broadly classified into seven functional groups (Supplementary Table 5) related to immune surveillance machinery. The average fold enrichment values of the enriched GO_mf_ categories are depicted in Figure 2C. Figure 2D shows that these functional groups share a considerable fraction of proteins among themselves. This indicates that the host targets are multifunctional and might have a role in inter-functional cross-talks.

#### GO_cc_ Enrichment Analysis

There were 42 statistically significant enriched GO_cc_ terms that were broadly classified into 15 groups (*P* < 0.05; Fisher’s exact test; multiple test correction using Benjamini–Hochberg method) (Supplementary Table 5). Figure 2E represents the fold enrichment values of these 15 GO_cc_ groups. It is observed from the aforementioned groups that *K. pneumoniae* target proteins localize at the elementary sites of host–pathogen interactions which include extracellular matrix (ECM) membrane ruffles and endocytic vesicles.

#### KEGG Pathway Enrichment Analysis

There were 26 statistically significant KEGG pathways among the inferred host targets (*P* < 0.05; Fisher’s exact test; multiple test correction using Benjamini–Hochberg method; Supplementary Table 6) which were further grouped into 14 subgroups. Figure 2G represents the average fold enrichment values of enriched KEGG pathways. Figure 2H shows that there are a significant number of proteins shared between the different enriched pathways. This implies that host targets could be involved in multiple pathways and have a role in pathway cross-talk. Among these, the NFKB signaling pathway is crucial for generating an immune response against any given pathogen. Moreover, the infectious disease pathway has several KEGG pathways targeted by other pathogens under it like HTLV-1, leishmaniasis, tuberculosis, etc. This again indicates the convergent targeting of metabolic pathways by a diverse range of individual pathogens.

From function enrichment analyses, it is inferred that *K. pneumoniae-*targeted host proteins are multifunctional and participate in diverse immune-related functions. Figures 2B,D,F,H show that there is a large degree of molecular overlap between enriched pathways, biological process and molecular functions. Hence, from these observations, we found it interesting to ask whether there is a group of common multifunctional proteins between each of these categories, i.e., GO_bp_, GO_mf_, and KEGG pathways. To address this possibility, we deployed a score-based functional prioritization strategy (see Supplementary Methods) and identified three multifunctional target proteins, HIF1A, STAT1, and TP53; TP53 encoding tumor suppressor protein P53 is a known master integrator of the host’s immune system (Madenspacher et al., 2013). HIF1A and STAT1 are all involved in physical interaction with P53 (Townsend et al., 2004; Obacz et al., 2013; Zhou et al., 2015). The interaction between P53 and HIF1A is fundamental for the induction of hypoxic pathways (Zhou et al., 2015). The hypoxic pathways are pivotal for regulating blood oxygen level which drops drastically in patients with pneumonia and sepsis (Hirota, 2015). It has also been observed that P53 regulates inflammation and autoimmunity by triggering proinflammatory cytokines owing to STAT1 activation (Campbell et al., 2012). Thus, P53 and its interaction partners could play a potential role in *K. pneumoniae-*mediated infection. This notion gets consolidated in subsequent downstream analyses.

### *K. pneumoniae* Preferentially Targets Inter-Complex or Intermodular Protein–Protein Interactions

**FIGURE 3:**
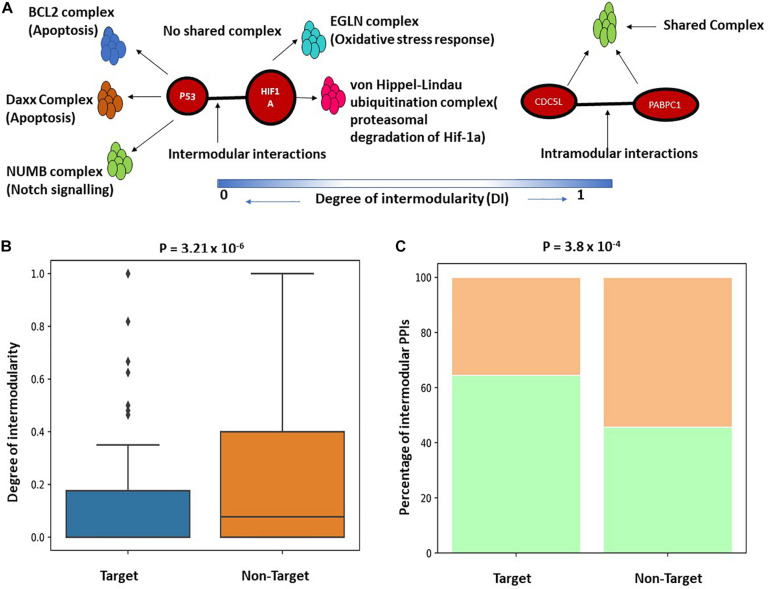
**(A)** Schematic representation of inter and intramodular interactions. Intermodular interactions are defined as the interactions between two proteins A and B if they participate in distinct functional complexes without having any shared protein complex between A and B (*DI* = 0). Intramodular interactions are defined as the interactions between protein A and B when there is at least one functional protein complex shared between A and B (*DI* = 1). **(B)** Boxplot showing a significant difference in the distribution of *DI* between targeted PPI and non-targeted PPI. *DI* is defined as the ratio of the number of shared protein complexes between two targeted and non-targeted interacting protein pairs to the total number of unique protein complexes in which A and B participate. **(C)** Stacked bar plot showing a higher percentage of intermodular interactions among targeted PPIs as compared to non-targeted PPIs.

The functional enrichment analysis indicates that *K. pneumoniae-*targeted host proteins are participated in multiple interrelated functions and are involved in molecular cross-talk between biological processes and immune-signaling pathways. To further establish this fact, we here studied the *K. pneumoniae-*targeted PPIs and interrogated whether the PPIs targeted by *K. pneumoniae* are intermodular. Intermodular protein interactions are those that arise between two proteins that never share any given protein complex between them and intramodular protein interactions are those that occur between two proteins that share at least one protein complex between each other (Song and Singh, 2013). For instance, in Figure 3A, P53 and HIF1A physically interact with each other in the human PPI network. Both the proteins P53 and HIF1A participate in distinct protein complexes without a single protein complex being shared between them. On the contrary, Figure 3B shows an intramodular or intra-complex interaction between protein CDC5L and PABPC1, both of which participate in the spliceosomal protein complex. To validate this hypothesis, we designed a brief computational workflow (Supplementary Figure 1A). We started with building the human PPI network by assembling experimentally established PPI data from two resources BioGRID and STRING (Supplementary Table 7). We next selected *K. pneumoniae-*targeted PPIs where both the proteins involved in interactions are targeted by KP. Next, we considered the largest connected components (LCC) of the aforementioned *K. pneumoniae-*targeted interactions. The LCC of the *K. pneumoniae-*targeted PPI network (TTPPI) consisted of 65 target proteins connected through 100 connections (Supplementary Table 7). To analyze the proportion of inter-complex interactions in this *K. pneumoniae-*targeted PPI network, we took two approaches: (i) we calculated the proportion of inter-complex interactions in the targeted network and compared it with non-targeted PPI network (Supplementary Table 7). Non-targeted PPI network is composed of PPIs that involve those proteins that are never targeted by *K. pneumoniae*. Figure 3C shows that the proportion of inter-complex interactions in *K. pneumoniae-*targeted network is way higher than non-targeted PPI network (61 connections out of 100 were identified to be inter-complex interactions which are approximately 61% of the total targeted connections). This consolidates our hypothesis that *K. pneumoniae* preferentially targets inter-complex PPIs as compared to intra-complex PPIs. (ii) We formulated the *DI* (see section “Materials and Methods”) of a PPI and compared the *DI* between targeted PPI and non-targeted PPI. The *DI* can be calculated as the number of shared protein complexes between two proteins divided by the total number of protein complexes in which the two proteins participate. It was observed that the *DI* of *K. pneumoniae-*targeted PPI network was significantly higher as compared to the non-targeted network (Figure 3B). This confirms our hypothesis that *K. pneumoniae* preferentially targets intermodular or inter-complex PPIs.

### P53 and CDC5L Form the Backbone of Cross Complex Interactions

In the previous section, we showed that *K. pneumoniae* preferentially targets inter-complex or intermodular PPIs. Supplementary Figure 1C shows that TTPPI has several *K. pneumoniae-*targeted, inter-complex interactions that could play an important physiological role in coordinating the functions of these protein complexes. Next, we extracted only the inter-complex connections from the TTPPI and derived a smaller network (ITTPPI) (Supplementary Table 7). Supplementary Figures 1B,C indicate that two proteins in the ITTPPI network play an influential role in maintaining network robustness as they participate in multiple inter-complex connections. These two proteins are P53 and CDC5L. P53 has the second largest degree or inter-complex connections in this network (K_p__53_ = 17; K_average_ = 5) while CDC5l ranks the highest influential node in the network with K of 21. Thus, both of these two proteins are important for maintaining molecular cross-talk between several interrelated functional complexes. For instance, P53 not only participates in different functional complexes related to apoptosis and DNA damage response but also forms unique 17 PPIs with different protein partners that participate in allied functional complexes like chromatin remodeling, immune signaling, and mRNA processing. Thus, coordination of these functional complexes, e.g., apoptosis and immune signaling, could be perturbed in *K. pneumoniae-*mediated infection via P53. The connections of CDC5L with its protein partner might help in the maintenance of molecular cross-talk between complexes like proteasomal degradation, chromatin modification, etc.

### The Potential Functional Cross-Talks That Might Be Perturbed by *K. pneumoniae*

**FIGURE 4:**
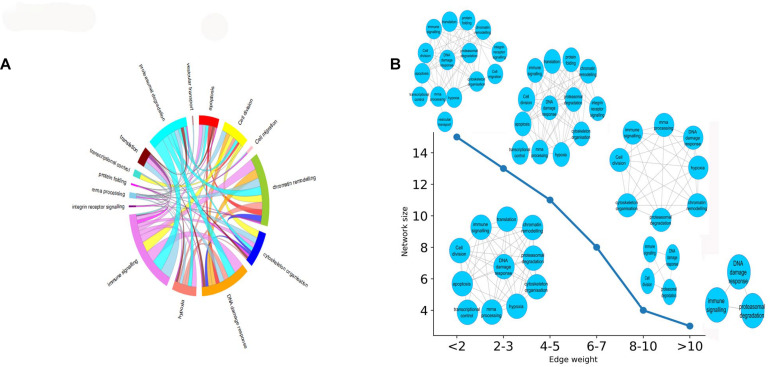
**(A)** Circos plot representing the functions or biological processes of protein complexes that are connected by intermodular PPIs targeted by KP. The width of the ribbons within the circus plot represents the number of host targets that mediate the above-mentioned inter-complex interactions, i.e., wider ribbons are indicative of a higher number of host target proteins while narrow ribbons indicate the existence fewer number of host target proteins mediating the given inter-complex interactions. **(B)** How the FFIN shrinks as we increase the edge weight of the FFIN network. The *X*-axis here represents the edge weight (measured as the no. of inter-functional connections, i.e., number of inter-complex PPIs) and the *Y*-axis represents the network size measured as the number of nodes.

In this section, we aimed to evaluate the functional impact of cross complex interactions disrupted by *K. pneumoniae*. Supplementary Table 8 enlists proteins targeted by *K. pneumoniae* along with the functional complexes in which they participate. These protein complexes were further grouped into 15 major classes according to their functions: (a) immune signaling, (b) chromatin modification, (c) DNA damage response, (d) apoptosis, (e) cytoskeleton organization, (f) proteasomal degradation, (g) transcriptional control, (h) translation, (i) integrin receptor signaling, (j) mRNA processing, (k) cell migration, (l) protein folding, (m) cell division, (n) vesicular transport, and (o) hypoxia. All of these functions are tightly associated with each other by multiple *K. pneumoniae-*targeted inter-complex PPIs. Figure 4A shows a circos diagram in which the width of the ribbons connecting each function represents the number of unique *K. pneumoniae-*targeted inter-complex interactions between given functions. For instance, there is a wide ribbon connecting immune signaling to proteasomal degradation which indicates that there are a large number of inter-complex connections between immune signaling complexes and proteasomal degradation machinery. This, in turn, points at the fact that proteasomal degradation machinery has multiple molecular cross-talks with immune signaling processes which is also well documented in literature (Kammerl and Meiners, 2016). Next, we constructed an FFIN from the aforementioned inter-complex interaction data where each node denotes a particular function or biological process, and the connection between them represents the number of inter-complex PPIs. Each edge connecting the nodes is assigned with a weight which is the sum of the number of inter-complex PPIs between the given functions or nodes. Figure 4B shows that as we gradually increase the stringency of the FFI in terms of edge weight, the network shrinks. At highly stringent edge weight, the network consists of only three nodes, i.e., immune signaling, proteasomal degradation, and mRNA processing. This hints at an important mechanism of immune regulation. It has been reported previously that immune regulators are often controlled or regulated by the process of proteasomal degradation (Hu and Sun, 2016). Hence, from these observations, we infer that *K. pneumoniae* might interfere with proteolytic degradation of immune system modulators like P53. This, in turn, shows that these three biological processes have the highest number of inter-complex interactions that are targeted by *K. pneumoniae*. At the lowest stringency, the network grows in size, and many other nodes are added including integrin receptor signaling, cell migration, apoptosis, hypoxia, or oxidative stress.

We have observed in the previous section that most of this inter complex interaction or functional cross-talks observed are mainly mediated by the master coordinator P53. It is also noticed (Supplementary Figure 1C) that *K. pneumoniae* targets the interaction of P53 with its protein partners. In the next stage of our analysis, we explored the impact of *K. pneumoniae* targets in the host regulatory network as well. In this connection, we identified different regulatory cascades that could mediate transcriptional response of host in *K. pneumoniae-*mediated sepsis. Here also, the role of P53 centric ITTPPI was prominent as we identified four pivotal *K. pneumoniae-*targeted TFs (TTFs) which are an integral part of ITTPPI (Supplementary Figure 1C) to be the major source of transcriptional response in human host.

### Identification of Key *K. pneumoniae-*Targeted TFs That Regulate Downstream Sepsis Responsive Genes and Their Connections With P53

**FIGURE 5:**
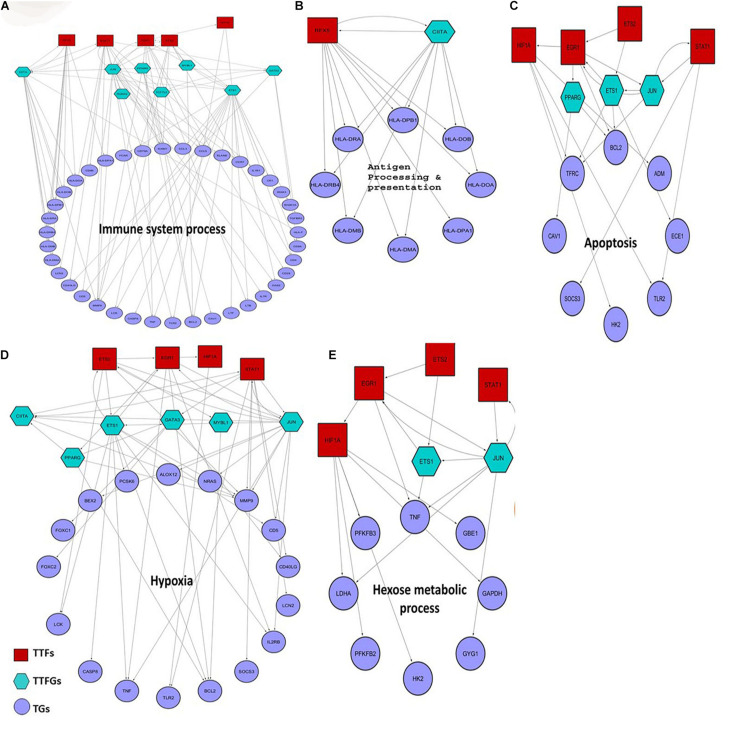
Small regulatory networks where key TFs are shown to target the different functional subset of DEGs in *K. pneumoniae* positive sepsis patients. The red squares indicate the TTFs, the sea-green hexagon represents TTFGs, and mauve circles represent the TGs. **(A–E)** The functional subsets of DEGs targeted by TTFs and TTFGs, i.e., immune system process, antigen processing and presentation, hypoxia, apoptosis, and hexose metabolic process, respectively.

Previous studies suggest that 5–20% of bacterial sepsis is caused by *K. pneumoniae*. Moreover, patients suffering from *K. pneumoniae-*mediated sepsis are often associated with poor prognosis and severe outcomes like death (Meatherall et al., 2009; Fukuyama et al., 2014). Hence, we find it interesting to investigate whether some of the inferred host targets in our study play an instrumental role in *K. pneumoniae-*mediated sepsis. To figure this out, we designed a four-step computational strategy (see Supplementary Figure 2), where in the first step, we assembled information on TFs and their target mRNAs (TGs) in humans from the public databases to build a raw regulatory network (RTRN) (Supplementary Table 9). In the second step, we derived a sepsis regulatory network (SRN) from RTRN by selecting only the genes in the RTRN that are found to be differentially expressed under sepsis conditions (DEGs). The complete list of DEGs in *K. pneumoniae* positive sepsis patients is provided in Supplementary Table 10. In the third step, we derived *K. pneumoniae* specific SRN or KPSRN where we retained only TFs that were targeted by *K. pneumoniae* (TTFs) and their downstream TFs (TTFGs) or TGs (Supplementary Table 9). The TGs in the KPSRN were functionally analyzed using GO analysis in the final step. The functional enrichment analysis using the TGs yielded five important functional groups which are: (a) antigen processing and presentation, (b) immune system process, (c) response to hypoxia, (d) hexose metabolic process, and (e) regulation of programmed cell death or apoptosis which are previously reported to be linked to the outcome and progression of septicemia (Träger et al., 2003; Noritomi et al., 2009; Chaudhry et al., 2013; Wu et al., 2017; Bouras et al., 2018). The detailed results of the GO analysis have been represented in Supplementary Table 11. As the aforementioned five functional groups are related to the sepsis outcome they are of utmost significance. However, there might be other significant functional groups (Supplementary Table 11) that are related to host’s response to pathogenic insult. One such group is genes linked to cation homeostasis. It has been reported earlier that bacterial pathogen like *K. pneumoniae* competes with the host to scavenge nutrients like trace cations especially iron and calcium (Palmer and Skaar, 2016). Therefore, metal ion homeostasis may not be directly associated with sepsis outcome or progression but is important for bacterial survival within the host. The TTFs and TTFGs involved in the regulation of each of these functional classes of proteins are represented in Figures 5A–E. Figures 5A–E show that there are four pivotal TTFs, ETS2, EGR1, STAT1, and HIF1A which targets four of the five downstream functional clusters including apoptosis, hexose metabolic process, hypoxia, and immune system process.

It is also interesting to note that these four TFs are all associated with P53 centric ITTPPI. The connections between each of these four TFs with P53 are elucidated in Supplementary Figure 1C. P53 and its contribution to pneumonia are well studied previously where it was shown that P53 regulates and coordinates the function and fate of phagocytes in mouse lungs (Madenspacher et al., 2013). In our study, we did not observe any significant changes in the expression level of P53 in sepsis patients as compared to healthy individuals. But, the involvement of interactors or regulators of P53 is observed in host response to *K. pneumoniae-*mediated sepsis. This hints at the fact that *K. pneumoniae* might perturb P53 and its interaction with close allies to cause sepsis in the host. Thus, the p53 centric ITTPPI obtained in PPI network analysis contains some of the interactor TFs of P53 which controls downstream regulation of functional DEGs in *K. pneumoniae* positive sepsis patients.

## Discussion

This study aims to decipher host–pathogen bio-molecular interactions that may shed light on the potential mechanisms of *K. pneumoniae* virulence within the host. Our study can be divided into four individual stages.

Stage I involves the identification of host targets using the interolog approach and subsequent screening of the inferred interactions by *in vivo* fitness, *in silico* prediction of GO term similarity, and secretion propensities. Interolog approaches of PPI prediction often yield false positives. To minimize retention of false positives in our dataset, we have deployed the three screening filters, i.e., *in vivo* fitness, GO term similarity, and secretion propensity. To date, the interolog approach has been widely used to predict bacteria–host PPI (Remmele et al., 2015; Kumar et al., 2019). This method has few limitations, for instance, the method is homology dependent and does not predict interactions between proteins that are strain-specific or lineage-specific. Second, it relies on experimentally validated bacteria–host PPI data which is used as a template to predict the potential PPIs. Currently, there is a dearth of experimentally validated bacteria–host PPI data as experimental methods used to infer PPIs are time-consuming and require a lot of efforts. Protein structure-based predictions of PHPPI are also used in many studies (Mariano and Wuchty, 2017). However, the lack of well-reviewed *K. pneumoniae* protein structures in publicly available databases limits the application of the structure-based method to identify potential KHPPIs. On the other hand, virulence effectors that are successfully deployed by the pathogen to hijack host immunity have high *in vivo* fitness cost upon deletion from pathogen genome (Crua Asensio et al., 2017). Thus, this method of prioritization using fitness enables us to analyze the effect of influential virulence effectors produced by *K. pneumoniae* on host’s physiology. Moreover, we have added two additional screening filters: (i) GO term similarity between the inferred homologs which predict the homologs obtained using reciprocal blast search are potentially true homologs and (ii) secretion propensity which assess the obtained *K. pneumoniae* proteins involved in interaction with the host are extracellular or are secreted at host–pathogen interface to carry out interaction with potential host targets. However, all the molecular targets inferred in this study are computationally predicted and need further experimental validations.

Stage II involves function enrichment analyses followed by functional prioritization protocol. The results of stage II analysis indicate that host targets are associated with immune surveillance machinery of host. This further hints at the hijacking of the host’s immune system by *K. pneumoniae*. Moreover, we obtained five most multifunctional proteins (based on multifunctionality scores) which are P53, HIF1A, and STAT (Figure 2I). All of these five multifunctional proteins are closely associated with P53 in terms of physical interaction or transcriptional regulation (Townsend et al., 2004; Rozan and El-Deiry, 2006; Obacz et al., 2013). P53 itself is a master coordinator of immune system (Lowe et al., 2013; Menendez et al., 2013; Levine, 2020) and is responsible for its effect in diverse functions and pathways (Junttila and Evan, 2009; Brooks and Gu, 2010) including its contribution to apoptosis and DNA damage response (Brooks and Gu, 2010). P53 has also been shown to cross-talk with hypoxic pathways which are pivotal in lung infection (Obacz et al., 2013; Zhou et al., 2015). This cross-talk takes place between P53 and the hypoxia-inducible factor, HIF-1 alpha, which is an inducer of hypoxic response in human (Greijer and van der Wall, 2004). On the other hand, P53 and STAT1 cooperate to induce cell death in response to DNA damage (Townsend et al., 2004). It has also been previously proposed that P53 is involved in TNF alpha-induced apoptosis (Rokhlin et al., 2000). In a nutshell, the two multifunctional proteins, i.e., HIF1A and STAT1, are all connected with P53 either through direct interaction or through transcriptional regulation. This gives an initial hint that P53 and its associated proteins could be highly influential targets of *K. pneumoniae*. But how P53 and its allies could be related to the physiological response of host during *K. pneumoniae* infection remains unidentified. To explore this, we have studied the human PPI network and transcriptional regulatory network in stage III and stage IV of our analyses.

In stage III, we mined the human PPI network to identify regions of the human PPI network enriched in host targets. We observed that *K. pneumoniae* targets part of the human PPI network (TTPPI) which is enriched in inter-complex or intermodular interactions (Figures 3C,D). P53 and CDC5L form the essential backbone of inter-complex interactions in the ITTPPI (Supplementary Figures 1B,C). An influential target of *K. pneumoniae* could be CDC5L. Role of CDC5L in cell cycle regulation, immune surveillance, and DNA damage response is well studied (Zhang et al., 2009; Mu et al., 2014). Thus, targeting CDC5L might perturb these physiological processes. On the other hand, the functional cross-talks targeted by *K. pneumoniae*, the majority of which are mediated by P53 and CDC5L, are enlisted in Figure 4B. Thus, P53 not only acts as the multifunctional protein but also a coordinator of several inter-complex or intermodular connections. The two most important intermodular connections targeted by *K. pneumoniae* are the connection between immune signaling process and proteasomal degradation (Zinngrebe et al., 2014; Hu and Sun, 2016; Kammerl and Meiners, 2016; Ebner et al., 2017; Etzioni et al., 2017) as well as immune signaling process and DNA damage response (Gasser and Raulet, 2006; Nakad and Schumacher, 2016; Bednarski and Sleckman, 2019) (Figure 4B). It has been reported earlier that proteasomal degradation is a key cellular process that regulates immune signaling events (Junttila and Evan, 2009; Kammerl and Meiners, 2016). Thus, disturbing the coordination between these two pivotal processes, i.e., immune signaling and proteasomal degradation, may lead to severe impairment of immune function. Next, the intermodular connections between DNA damage response and immune signaling process are also targeted by *K. pneumoniae*. Immune signaling networks often form alliances with DNA damage response system (Gasser and Raulet, 2006; Nakad and Schumacher, 2016). The disruption of coordination between these two pivotal processes, i.e., immune signaling and DNA damage response, can also lead to severe immune defects (Gasser and Raulet, 2006; Nakad and Schumacher, 2016; Bednarski and Sleckman, 2019). Therefore, in a nutshell, we infer that *K. pneumoniae* targets several intermodular connections by targeting the P53 interactome. The disruption of coordination between these functions can severely impact the immune response of the host. Furthermore, in our next stage, we have considered *K. pneumoniae-*mediated sepsis as a disease model and build a transcriptional regulatory network based on DEGs in *K. pneumoniae* positive sepsis which indicates the role of P53 interactome in host’s transcriptional response to sepsis.

In stage IV, we compared host’s transcriptome under sepsis and healthy conditions, respectively, and identified DEGs which are the major source of transcriptional response to *K. pneumoniae-*mediated sepsis. Using our computational work flow (Supplementary Figure 2), we found four pivotal differentially expressed TFs (EGR1, HIF1A, STAT1, and ETS2) that are part of the P53 interactome, and control downstream genes which were further functionally grouped into five interrelated functional categories including apoptosis, antigen presentation and processing, response to hypoxia, immune system, and carbohydrate metabolism (Figure 5). Each of these functions is correlated with the host’s physiological response during sepsis and therefore is of utmost importance. We would therefore concentrate on these five functional subsets of genes. These five functional gene groups are important for adaptive as well as maladaptive response of host to *K. pneumoniae* infection. However, the main caveat of using host’s transcriptomic data is that it does not differentiate between *K. pneumoniae* specific transcriptional response and general response to bacterial infections. There are many common infection specific pathways that are activated during other pathogenic insult as well as *K. pneumoniae* specific infection. For instance, the antigen presentation and processing pathways are activated in response to other pathogens as well in *K. pneumoniae* specific infection. Thus, *K. pneumoniae* specific response cannot be captured using the current methodology. But it is evident that many of the *K. pneumoniae-*targeted host proteins participate in pathways that are activated during other pathogenic infections as well and therefore are not *K. pneumoniae* specific.

Starting with antigen processing and presentation, it was reported that severe defects in antigen processing and presentation due to a reduced expression level of HLA genes are a common scenario in sepsis (Meisel et al., 2009; Wu et al., 2017). CIITA and RFX5 co-transactivates the HLA genes (Kern et al., 1995). In our analysis, we found that *K. pneumoniae* TTF, RFX5 severely downregulated in *K. pneumoniae* positive sepsis patients. Also, we found that both RFX5 and CIITA cooperate and regulate the HLA genes responsible for proper antigen presentation (Figure 5B). Sepsis is primarily caused by overactivation of the immune system leading to the production of inflammatory cytokines including interleukins and tumor necrosis factor (TNF) (Chaudhry et al., 2013; Schulte et al., 2013). Moreover, TNF is a multifunctional cytokine whose role in apoptosis and hexose metabolism is also reported (Gupta and Gollapudi, 2005; Remels et al., 2015). In our results, we observed that *K. pneumoniae* targets ETS2 whose expression has been reported to correlate with TNF alpha production and inflammation in endothelial cells (Wei et al., 2004; Brooks and Gu, 2010; Remels et al., 2015). Consistent with this observation, we in our study also found an important role of ETS2 TFs in the overproduction of TNF (Figures 5A,C–E). Apart from immunological alterations, metabolic alterations also take place in sepsis (Träger et al., 2003; Carré and Singer, 2008; Noritomi et al., 2009; van Wyngene et al., 2018). For instance, hyperglycemia or high glucose level in the blood often correlates with the severity of sepsis (van Wyngene et al., 2018). Hyperglycemia could be a result of inefficient glucose immobilization in the blood of sepsis patients due to reduced expression of glycolytic enzymes (van Wyngene et al., 2018). Here in our analysis, we identified a group of glucose metabolizing genes including HK2 (hexokinase) and PFKB2 (phospho-fructokinase 2) which are regulated by HIF1A another P53 interactor and a *K. pneumoniae* target. HIF1A and EGR1 are also found to jointly regulate hypoxic genes like TFRC (Transferrin receptors) (Tacchini et al., 1999; Holden et al., 2016) and Caveolins (CAV1) (Bourseau-Guilmain et al., 2016). Moreover, as previously mentioned, HIF1A, ETS2, EGR1, and STAT1 are all associated with deregulation of each of these functional clusters. Four of these TFs have a close association with P53 as well in the ITTPPI (Supplementary Figure 1C).

Hence, we in this study provide several lines of indications that targeting of P53 and the P53 centric interactome (ITTPPI) might result in inducing host’s transcriptional response against *K. pneumoniae* infections. Thus, targeting of P53 centric interactome might be one of the significant strategies adopted by *K. pneumoniae* to subvert host biomolecular machinery.

## Data Availability Statement

The original contributions presented in the study are included in the article/Supplementary Material. Further inquiries can be directed to the corresponding author/s.

## Author Contributions

DS and SK designed and conceptualized the study. DS developed and executed various computational workflows, collected the data, did the literature survey, and wrote the manuscript. SK edited the manuscript, supervised the entire study, and provided guidance. Both authors contributed to the article and approved the submitted version.

## Conflict of Interest

The authors declare that the research was conducted in the absence of any commercial or financial relationships that could be construed as a potential conflict of interest.

## Footnotes

1 https://hpidb.igbb.msstate.edu/

2 https://string-db.org/

3 https://thebiogrid.org/

4 http://mips.helmholtz-muenchen.de/corum/

5 http://www.regnetworkweb.org/

6 https://www.grnpedia.org/trrust/

## Supplementary Material

Supplementary Figure 1 **(A)** Brief workflow of the PPI network analysis describing the major steps of the methodology used to obtain the TTPPI. **(B)** The degree distribution of each of the nodes in the intermodular TTPPI network showing a high degree of P53 (K = 17) and CDC5L (K = 21). **(C)** The ITTPPI comprising of only intermodular interactions between *K. pneumoniae-*targeted host proteins. The hexagonal node in the ITTPPI network denotes the hub proteins P53 and CDC5L. The red rectangular nodes in the ITTPPI network are the TTFs or key regulators responsible for genetic dysregulation in *K. pneumoniae* positive sepsis patients.

Supplementary Figure 2 Computational workflow showing stepwise regulatory network analyses.
