# Supplementary figures and images for: A Molecular Interaction Map of Klebsiella pneumoniae and Its Human Host Reveals Potential Mechanisms of Host Cell Subversion
Source: Front Microbiol. 2021 Feb 18;12:613067. doi: 10.3389/fmicb.2021.613067 (PMC7930833; doi:10.3389/fmicb.2021.613067)

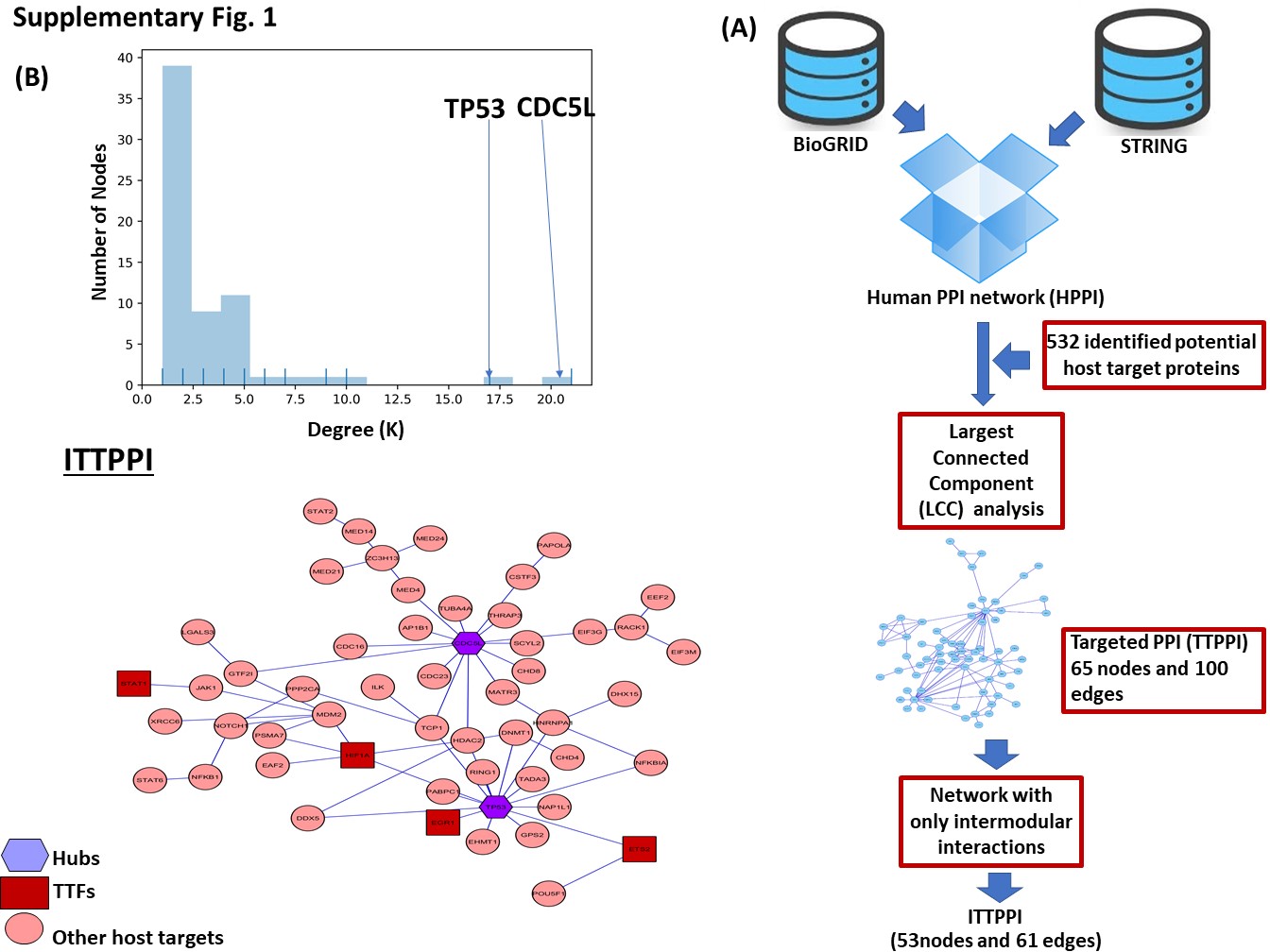

Supplement: Supplementary Figure 1 — (A) Brief workflow of the PPI network analysis describing the major steps of the methodology used to obtain the TTPPI. (B) The degree distribution of each of the nodes in the intermodular TTPPI network showing a high degree of P53 (K = 17) and CDC5L (K = 21). (C) The ITTPPI comprising of only intermodular interactions between K. pneumoniae-targeted host proteins. The hexagonal node in the ITTPPI network denotes the hub proteins P53 and CDC5L. The red rectangular nodes in the ITTPPI network are the TTFs or key regulators responsible for genetic dysregulation in K. pneumoniae positive sepsis patients. [file Image_1.jpg]

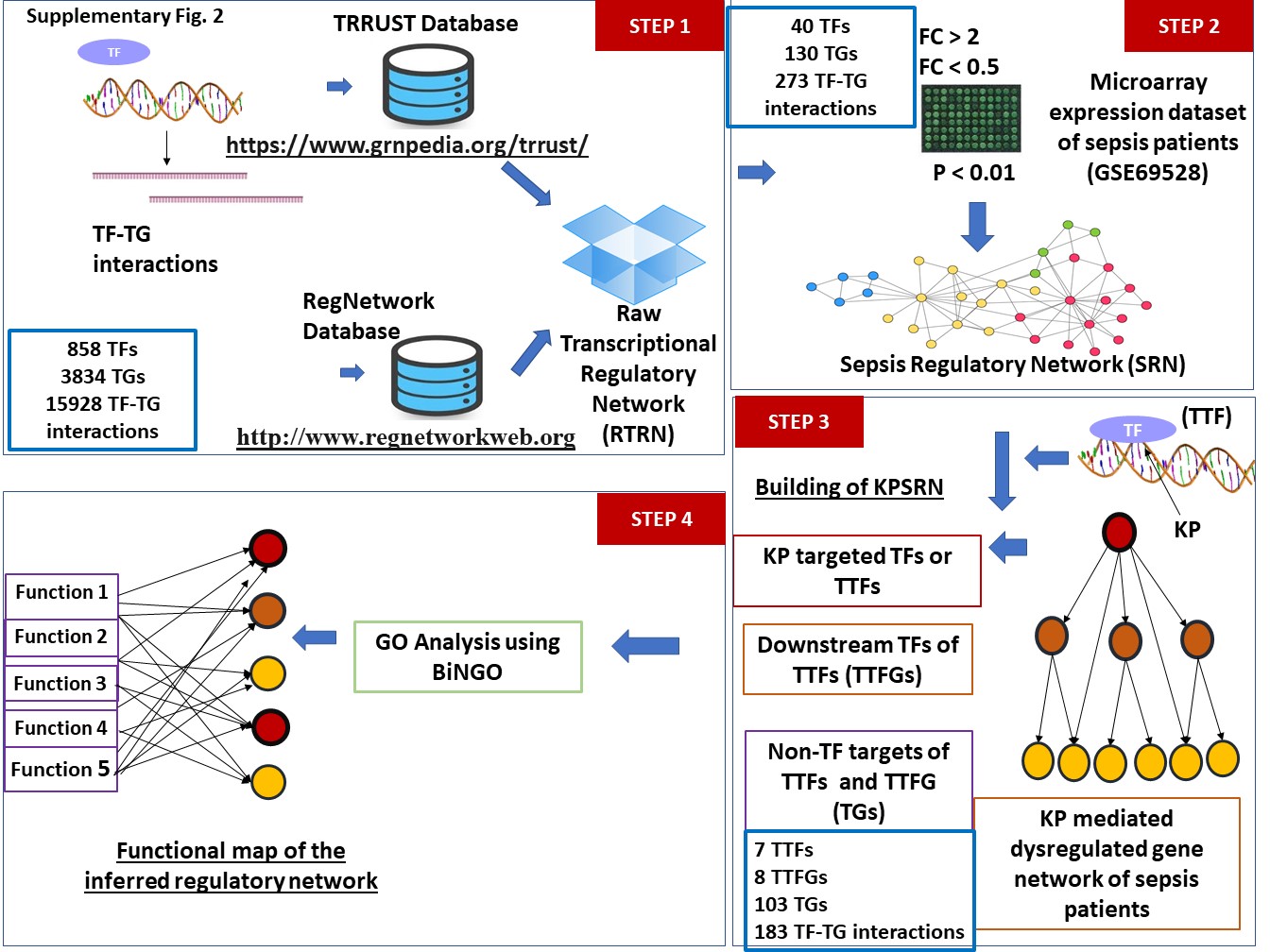

Supplement: Supplementary Figure 2 — Computational workflow showing stepwise regulatory network analyses. [file Image_2.jpg]
