# Supplementary material for: A Molecular Interaction Map of Klebsiella pneumoniae and Its Human Host Reveals Potential Mechanisms of Host Cell Subversion
Source: Front Microbiol. 2021 Feb 18;12:613067. doi: 10.3389/fmicb.2021.613067 (PMC7930833; doi:10.3389/fmicb.2021.613067)
Supplement: Supplementary file 5 [file Table_3.docx]

**Supplementary Table 3: List of four pathogen genomes which yielded significant hit upon reciprocal protein blast against *K. pneumoniae* proteins. The Table also represents the number of non-homologous protein pairs obtained for each pathogen genomes.**

| **Name of Pathogen species** | **NCBI Taxonomy ID** | **Number of non-homologous protein pairs against K. pneumoniae proteins** |
| --- | --- | --- |
| ***Yersinia pestis*** | **632** | **2200** |
| ***Bacillus anthracis*** | **1392** | **3781** |
| ***Francisella tularensis*** | **263** | **4256** |
| ***Escherichia coli K12*** | **83333** | **1120** |
